# Distribution, Sources and Risk Assessment of Polychlorinated Biphenyls in Sediments from Beiluo River

**DOI:** 10.3390/toxics11020139

**Published:** 2023-01-31

**Authors:** Linrong Han, Chao Chang, Shiwei Yan, Chengkai Qu, Yulu Tian, Jipu Guo, Jiahua Guo

**Affiliations:** 1 Shaanxi Key Laboratory of Earth Surface System and Environmental Carrying Capacity, College of Urban and Environmental Sciences, Northwest University, Xi’an 710127, China; 2 State Key Laboratory of Biogeology and Environmental Geology, School of Environmental Studies, China University of Geosciences, Wuhan 430074, China; 3 State Grid Shaanxi Electric Power Research Institute, Xi’an 710100, China

**Keywords:** positive matrix factorisation, ecological risk assessment, sediments, identification of sources, polychlorinated biphenyls

## Abstract

The distribution and source of polychlorinated biphenyls (PCBs) pollution in the Beiluo River, the secondary tributary of the Yellow River, still remain unclear. With the purpose of determining the distribution, origins, and pollution levels of PCBs and their consequences on ecological risks, the concentrations of 27 PCBs at 17 locations in the sediments of the Beiluo River were examined in this study. The results showed that the mass concentrations of ∑PCBs in the sediment ranged from 0.12 to 1.25 ng∙g^−1^ (DW), with the highest point at sampling site B13 downstream of the river. Compared to most river sediments, both domestically and internationally, the concentration of PCBs in the sediment of the Beiluo River was at a low level, with 10-PCB and 6-PCB as the main components, indicating that the PCBs that are difficult to volatilise and degrade are more likely to remain in the sediment. The origins of PCBs in the sediments of the Beiluo River were examined by using positive matrix factorisation (PMF). The results revealed that the contamination of PCBs in the sediments of the Beiluo River mainly resulted from industrial emissions, technical PCB mixtures, and coal and wood combustion. The results of the ecological risk assessment indicated that PCBs in the sediments of the study area rarely contribute to adverse biological effects and the potential low risk to the environment.

## 1. Introduction

In recent years, owing to rapidly growing populations and industrialisation, emissions of environmental pollutants have been increasing. Polychlorinated biphenyls (PCBs) are widely applied in capacitors, transformers coating, lubricants and other industrial products due to their excellent thermal stability, insulating properties and flame retardant [1]. Studies over the past 30 years have found PCBs in collected samples of seawater, river water and sediment collected worldwide [2]. PCB levels in fish samples were positively correlated with PCBs in seawater and surface sediments [3]. These PCBs are able to enter into human body through a variety of routes, including inhalation, dermal contact and food [4]. As a result, PCBs are on the United States Environmental Protection Agency’s list of priority pollutants and are listed as one of the persistent organic pollutants (POPs) under the Stockholm Convention [5].

From 1965 to 1980, more than 10,000 tons of PCBs were produced in China [6], and afterwards these substances had been banned. However, the leakage from such equipment has been reported, giving rise to a more serious hazard in some areas [7,8]. The pathways of PCBs entering into the riverine environment include domestic and industrial wastewater [9], non-point resource runoff and direct waste dumping. Due to the low solubility of PCBs in water, the Octanol-Water Partition Coefficient (Kow) is high; that is, its adsorption coefficient in sediments and enrichment factor in aquatic organisms are high. It has been suggested that PCBs bind with particulate matter in aquatic environments and eventually accumulate in underlying sediments [10]. PCBs can also enter the atmosphere through volatilisation during use and processing and can then be transferred to lakes and oceans through dry and wet settlement [11].

The Beiluo River (107°39′–110°16′ N, 35°24′–37°15′ E) has a watershed area covering an area of 26,532.68 km^2^ and is 680 km long in Shaanxi Province. Moreover, it is crucial for the surrounding agricultural activities and industrial advancement [12]. The number of studies associated with PCBs in the Chinese environment has increased substantially on account of a series of global initiatives on organic pollutants and the growing concern regarding the risks these chemicals pose to human health and the environment [13]. The natural system of the Beiluo River has long been threatened by industrial wastewater and oil spills, which have exerted a negative impact on public health and the economic development of the surrounding environment [14]. There have not been many studies on the sources and ecological risk assessment of PCBs in the past. Therefore, it is urgent to fill in the information gap concerning the spatial distribution of PCBs in the North River. The aim of this study is to determine the possible sources of PCBs in sediments and to assess the corresponding ecological risks.

## 2. Materials and Methods

### 2.1. Study Area and Sampling

**Figure 1:**
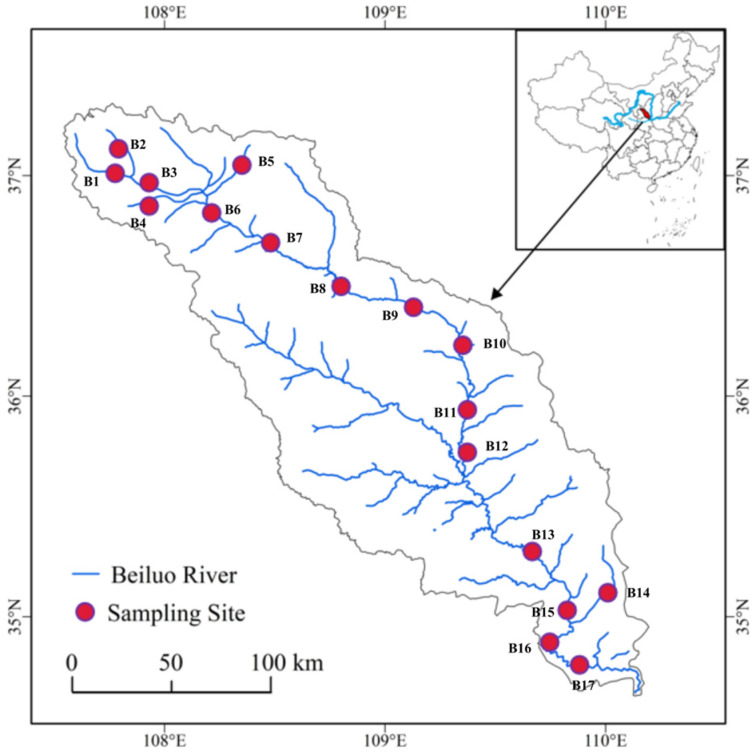
Study area with sampling sites marked with red dots.

Samples were taken from the Beiluo River (Shaanxi, China). The main stream of the North River is 680.3 km long, flowing from the northwest to the southeast through the Guanzhong Plain and Loess Plateau. A total of 17 sampling points were set up from upstream to downstream of the river based on the topography of the Beiluo River and historical data in June 2021 (Figure 1). Three sediment samples were taken from each site, each of which was approximately 2 kg. Sediment was collected from a depth of 0 to 10 cm using a grab mud collector sediment. Samples were placed in polyethylene plastic bags and transported back to the laboratory in a foam box with ice packs, stored at –4 °C until analysis.

### 2.2. Sample Pre-Treatment and Instrumental Analysis

The sediment samples were processed according to the methods in Soil and Sediment Determination of Polychlorinated Biphenyls (PCBs)—Gas Chromatography mass spectrometry (HJ 743-2015) [15]. The specific operation steps are provided in the Supplementary Materials (2-2). Gas chromatography (GC, Agilent 6890N/5975 MSD, USA) was used, which was equipped with an HP-5MS capillary column (30 m × 0.25 mm × 0.25 mm) and an electron impact (El) ionization source (GC-MS/MS 7000D). The carrier gas used was high-purity helium at a flow rate of 1.2 mL/min. For PCBs, the initial oven temperature was 80 °C for 1 min, followed by increases of 200 °C at 15 °C/min, 260 °C at 4 °C/min, 290 °C at 20 °C/min, held for 2 min, and then an immediate increase reached 320 °C with an injection volume of 1 μL. The electron energy was 70 eV, the ion source temperature was 230 °C, and the transmission line temperature was 280 °C. Finally, the concentration of PCBs in the samples from each sampling site was recorded. All analytical procedures were subject to strict quality assurance and quality control. Prior to extraction, each sample was added to labeled recovery standards (TCMX, PCB-65, and PCB-155) for identification [16]. The average recoveries ranged from 33.52% to 97.55% for TCMX, 47.24% to 108.79% for PCB65, and 27.91% to 113.59% for PCB155 [17]. While in our study, the average recoveries of TCMX, PCB65 and PCB155 were 52.76%, 51.63% and 56.74%. The result shows that the experiment is feasible to treat the samples.

### 2.3. Ecological Risk Assessment Methods

Based on relevant historical testing data, we chose three methods: the sediment quality guideline (SQG), toxicity equivalents (TEQs) and the Potential Ecological Risk Index Method (RI).

The SQG is a Canadian sediment environmental quality standard evaluation method that provides interim sediment quality/guidelines values (ISQG) and the probable effect level (PEL). When PCB concentrations are below the ISQG (ISQG = 25.1 ng∙g^−1^), PCB exposure to organisms is acceptable and rarely results in negative biological effects. When PCB concentrations are between the ISQG and PEL (PEL = 189 ng∙g^−1^), PCB exposure to organisms is a threat and may lead to negative biological effects. When PCB concentrations are above the PEL, PCBs pose a serious threat to the exposure of organisms, and the potential for negative biological effects is high. As there are no risk assessment criteria for PCBs in sediments in China, and the database of PCB toxicity to biological species in various regions of China is not well developed, there may be some errors in evaluating the risk of PCB contamination in river sediments in China based on this standard indicator. Therefore, researchers often choose multiple methods to jointly determine the risk assessment of PCBs.

TEQs can assess the ecological risk of PCBs [18]. The TEQs for PCBs in samples were calculated by using toxicity equivalence factors (WHO-TEFs) [19]. The total TEQ was calculated by multiplying the actual concentration of PCBs in the environmental sample by the corresponding toxic equivalency factor.

TEQ = ∑^N^_i_Ci × TEFi (1)

TEQ is the total equivalent (ng∙g^−1^). Ci is the detected PCB amounts in the sediment sample (ng∙g^−1^). TEF is the toxic equivalency factor of certain PCBs (the TEF values were taken from the WHO, 2005).

The Potential Ecological Risk Index reflects not only the impact of each pollutant in a particular environment but also the combined impact of multiple pollutants. This study was conducted by using quantitative methods to classify the level of potential ecological risk.

RI = ∑E^i^_r_ (2)

E^i^_r_ = T^i^ × C^i^_f_ (3)

C^i^_f_ = C^i^/C^i^_n_ (4)

C^i^_f_ is the pollution index of a pollutant; C^i^ is the measured concentrations of contaminants in the sediment; C^i^_n_ is the global contaminant levels in pre-industrial sediments (the PCBs in this paper are taken to be 0.01 mg/kg); E^i^_r_ is the potential risk parameters for individual pollutants; and T^i^ is the toxic response parameters for individual pollutants (T^i^ = 40). In this study, RI is equal to E^i^_r_.

The risk levels corresponding to the indices of individual risk factors are as follows: E^i^_r_ < 40 is a minor ecological hazard; 40 ≤ E^i^_r_ < 80 is a medium ecological hazard; 80 ≤ E^i^_r_ < 160 is a strong ecological hazard; 160 ≤ E^i^_r_ < 320 is a very strong ecological hazard; E^i^_r_ ≥ 320 is a pole-strength ecological hazard.

In this study, it was found that SQE can only describe the potential for negative biological impacts of sediments more roughly and cannot assess the integrated ecological riskiness of pollutants, whereas the TEQ method quantitatively assesses the integrated eco-toxicity under multiple pollutants by calculating the TEQ values of individual pollutants, and the RI method integrates the ecological risk level on the basis of the above. That is, it makes up for the low accuracy deficiency of the SQE method, reduces the unreliability of the TEQ method in predicting eco-toxicity singly and improves the accuracy of the ecological risk assessment.

### 2.4. Statistical Analyses

All statistical analyses were performed using Microsoft Excel. PCB sources were identified using Positive Matrix Factorization (PMF) 5.0 software (EPA, USA). The = PMF model error estimation results are within a reasonable range (Table S1). Two input files were required for the PMF analysis, the concentration and its uncertainty, and a method 5.0 user guide for calculating the uncertainty of individual contaminants was prepared based on the PMF. The level of a specific PCB was determined to be 1/2 that of the MDL at the method detection limit (MDL). The model was applied to the PCB data set and run at least 20 times, and the results were gathered/collected. The spatial distribution of the sampled points was mapped using the Arcgis 10.2 program (ESRI Inc., Redlands, CA, USA). The bar graphs were drawn using Prism 8.0 software.

## 3. Result and Discussion

### 3.1. PCB Concentrations in Sediments

**Figure 2:**
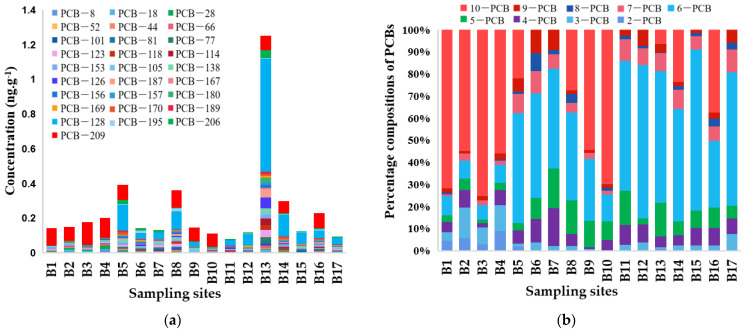
(**a**) PCBs concentrations in sediment sampling points (**b**) Contribution of PCBS homolog-ous spectral concentrations.

PCB concentrations in the sediments varied significantly between sampling locations (Figure 2a). PCBs were detected at each of the 17 sampling sites, but there was a large variation in PCBs at different locations. Among them, sampling points B13, B5 and B8 in the downstream and upstream parts of the Beiluo River had higher PCB concentrations of 1.25 ng∙g^−1^, 0.39 ng∙g^−1^ and 0.36 ng∙g^−1^, respectively. In addition, B9-B11 in the middle part of the river had the lowest PCB levels (Figure 1 and Figure 2a). This may be due to the upstream and downstream being closer to areas with specific sources [20].

The concentrations of PCBs in the sediments ranged from 0.12 to 1.25 ng∙g^−1^, with a mean value of approximately 0.27 ng∙g^−1^. The concentrations of PCB-128 and PCB-209 were much higher than others, accounting for 30.87% and 26.86% of the total PCB concentrations, respectively (Table S2).

By comparing the total PCB concentrations in the river and marine systems in other studies at home and abroad, it was found that the PCB concentrations in the sediments of the Beiluo River were relatively low (Table 1). The comparative results provide some indication of the extent and compositional characteristics of the contamination in the sediments, although there are differences in time, geography and detection methods among studies. The contamination characteristics of PCBs in sediments could reflect human activities, industrial and commercial development processes and the control and regulation of organic pollutants. Some studies have found positive correlations between the sources of contamination in sediments and human activities and industrial zones: The Pearl River Delta (16.15–477.85 ng∙g^−1^), Hooghly River (0.28–7.7 ng∙g^−1^) and Turag River (1.68–10.6 ng∙g^−1^). In addition, the results of Delaware River Estuary (18.1–136.8 ng∙g^−1^) and the Sanya River (1.75–92.75 ng∙g^−1^) demonstrated that factors such as current velocity, topographic conditions and total organic carbon content are linked to changes in the transport, distribution and adsorption of pollutants, which in turn affected the number of PCBs in the sediment.

The 27 PCBs were classified into different homologous groups (Figure 2b, Table S3), and it was found that 10-PCB and 6-PCB accounted for the largest proportion of PCBs at all sampling sites, which reached 81% and 63%, respectively. The overall proportion of high-chlorinated PCBs (>5Cl) was higher than low-chlorinated PCBs (<5Cl). In addition to the 6-PCBs, which contained the most PCBs, this may be because the degradation rate of PCBs decreased with increasing chlorine content [29]. Perchlorinated biphenyls were transported over a short distance and thus accumulated near the source [30], whereas lower chlorinated biphenyls were globally transported by atmospheric dry and wet deposition because of their higher vapour pressure [31]. It has also been shown that perchlorinated biphenyls were more lipophilic than lower-chlorinated biphenyls and were more likely to accumulate in the particulate phase and in sediments, migrating further with the tide [32].

### 3.2. Ecological Risk Assessment of Sediment PCBs

PCBs are a kind of industrial product of which no known natural sources have been identified. Some researchers have suggested that PCBs in the aquatic environment are mainly derived from land runoff and food chain transfer [33]. PCBs are volatile and are easily displaceable during water–atmosphere exchange, and these lost PCBs can be deposited back into rivers and land surfaces through deposition in the water column and runoff [34]. Thereafter, sediments store large amounts of organic pollutants and have a continuous environmental effect. A lot of research has been conducted overseas on the environmental risks of sediments and risk criteria have been established for some sediments [35,36]. In this study, we chose the sediment quality benchmark method (SQGs), potential ecological risk index (RI) and toxic equivalents (TEQs) to evaluate the pollution status of PCBs in the sediments of the Beiluo River.

According to the SQG method [33], the total sediment mass concentrations of PCBs at all 17 sampling sites in the Beiluo River were below the ISQG (interim sediment quality guideline) value for standard river sediment (25.1 ng·g^−1^), indicating that the exposure of PCBs to organisms in the sediment of this river section was acceptable and rarely caused negative biological effects [37].

The Potential Ecological Risk Index method was proposed by Swedish scientists in 1980 [38]. The method is based on quantifying the degree of potential ecological risk through the potential risk parameter (Er) (Table 2). In other words, the referenced concentration value of PCBs in sediment is 10 μg·g^−1^. Therefore, it is a potential ecological risk that the concentration value of PCBs is equal to or greater than this value at the sampling site. The results revealed that the potential risk parameter was <40 for sediments at all sampling sites, and the potential risk level was defined as minor, which is consistent with the results derived from the SQGs.

TEF values were originally used to assess the toxicity of abiotic samples to humans [39]. However, in recent years, this method has been applied to assess the toxicity of various environmental samples, including sediment samples [34]. Based on the structure, chemical composition and physiological consequences of PCBs, PCBs can be divided into two categories: dioxin-like and non-dioxin-like. Dioxin-like PCBs (DL-PCBs) are a class of very harmful by-products of industrial processes, similar to prototype dioxins. The TEQ of 12 DL-PCBs accounted for 18.27% of the total PCBs. The concentrations ranged from 0.0008 to 9.66 pg∙g^−1^, with a mean value of 0.93 pg·g^−1^ (Table 3). Referring to the ‘high risk to sensitive species’ (HR = 25 pg·g^−1^), it can be noticed that none of the PCBs-TEQs exceeded the HR limit at any of the sampling sites, indicating the low ecological risk of DL-PCBs in the sediment. Compared with domestic and international studies, the DL-PCBs-TEQs in the sediments of the Beiluo River were at a low contamination level, much lower than that of the Yamuna River [40], Dagu Drainage River [41], Nanpaiwu River [42], East River [43] and Lake Victoria [44].

The ecological risk evaluation of PCBs in the sediments of the Beiluo River based on the above three methods showed that there was slight contamination of PCBs in 17 sampling sites, which rarely triggered negative biological effects and posed a low potential risk to the environment. This may also have a lot to do with local hydrogeological and geomorphological conditions, the degree of social development and the level of government management [45]. Overall, although the content of PCBs in all sampling sites was within the safe range, the long-term accumulation effects still need attention. In the case of the Beiluo River, appropriate measures should be taken to prevent the deterioration of the sediment environment so as to reasonably plan industrial construction and strengthen production management. In addition, it is necessary to establish relevant laws and regulations and set up the most basic leakage prevention measures.

### 3.3. Source Identification of PCBs

**Figure 3:**
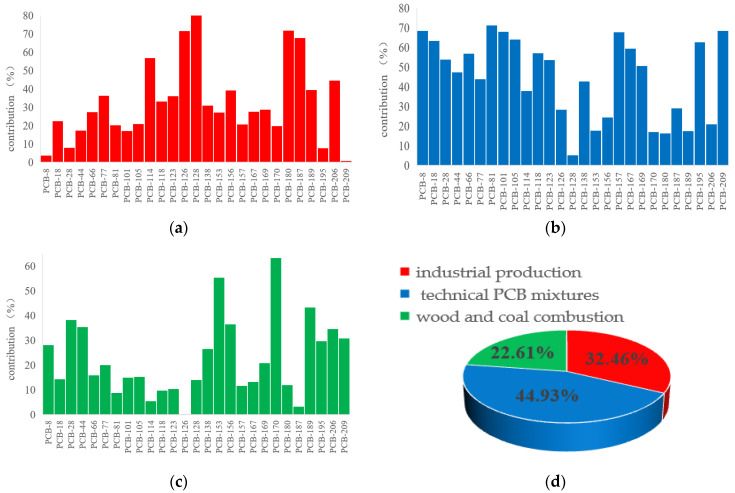
Positive matrix factorisation source profiles of PCBs (**a**–**c**). The bars represent the contribution of each factor to the corresponding PCB. (**d**) The pie chart shows the contribution of each factor to the total PCBs.

The distribution characteristics of PCBs in environmental media are usually related to their origin, transport and other factors in the environment. Two to eight different factors were selected for analysis using PMF until the most suitable solution within the margin of error was obtained. In this study, the sources of PCB contamination were finally identified as three relevant factors (Figure 3). The contribution ratio of these three factors to the total PCB sources was 32.46% (industrial production), 44.93% (technological PCB mixtures) and 22.61% (combustion of coal and wood) (Figure 3d).

The first factor mainly included PCB-128, PCB-126, PCB-180, PCB-187, PCB-114 and PCB-206, which all accounted for over 44.6% of Factor 1 (Figure 3a). It was found in previous studies that PCB-128 was a homologue of combustion markers [46], PCB-126 was at relatively high levels in products origin from high-temperature industrial processes such as smelting and the regeneration of non-ferrous metals [47], and PCB-114 was mainly used as a coating additive [48]. However, in PCB-126, 206 were thought to be formed during the production of various pigments, speculating that these PCBs may originate from the discharge of industrial effluents such as pigments and paints [49,50]. PCB-206 has also been detected in the production plants of titanium dioxide and titanium tetrachloride [51].

The source characteristics of these PCBs all suggest that the first factor might be potentially associated with industrial production. They are usually presented as by-products of industrial production processes, which are then released into the environment as waste gas, sludge or wastewater. In particular, after 1970, the country formulated a series of economic support policies for the western region, because of which the rich oil and coal resources in the upper Beiluo River region have witnessed a rapid development of industrial and mining industries [52]. The PCBs from these industrial fluids then accumulated in the environment through leakage and combustion. According to the survey, the industrial production part of the unintentional release of PCBs in China’s industrial production include high-temperature cement production, steel sintering, electric furnace steel making, metal smelting and waste incineration [53].

The second factor mainly includes PCB-81, PCB-101, PCB-157, PCB-8, PCB-209 and PCB-105, and all of these PCBs accounted for more than 61.32% of Factor 2 (Figure 3b). Among them, PCB-81, PCB-105, PCB-8 and PCB-101 are the main components of technical PCB mixtures [54], such as Arolcol 1242, Arolcol 125 and Kanechlor 600. Most of these PCBs are highly chlorinated PCBs, which are commonly used in the production of pigments and are considered to be an intentional source of PCBs [55]. Furthermore, the country’s total pigment production accounts for 70% of global production and generates a large amount of PCBs [56]. Therefore, this factor can be attributed to the technical PCB mixtures emitted during the production process.

The main PCBs in the third factor were PCB-170, PCB-153, PCB-189, PCB-28, PCB-156 and PCB-44, accounting for more than 43.56% of the total (Figure 3c). Previous research had found that PCB-156 and PCB-170 were mainly derived from wood and coal combustion [57]. Kim also proposed PCB-189 as a combustion marker homolog [46]. Therefore, the third factor is likely dominated by coal carbon and wood combustion. According to the survey, the PCBs produced from wood combustion mainly originate from domestic heating, the burning of surplus crops and forest fires, while the main sources of coal combustion are industrial production and domestic human needs [58]. The study area is a traditional coal and oil industrial zone [13]. Thus, coal proves to be an essential source of energy for the local industry. In addition, the main local crops are corn and soybeans, and the burning of straw crops is widespread in the local area.

Between 1965 and 1974, China produced about 10,000 tons of PCBs, which were used in capacitor-impregnating fluids, insulating oils and coating additives [59]. In the 1980s, the manufacture and commercial use of PCBs was banned, and most of the old electrical installations were sealed and placed in underground caverns [60]. Therefore, the possible sources and routes of contaminants still need to be investigated in greater depth to explore whether the sources of contamination are historical or persistent [61,62]. In addition, with changes in land use practices, large amounts of PCBs are released from environmental surfaces, such as soil and vegetation, into sedimentary systems [2]. In the sediments of different rivers, either domestic or abroad, these PCBs are transported over long distances by atmospheric or surface runoff, regardless of their origin [63]. During transport, different PCBs encounter different environmental fates. As a result, the composition of PCBs in sediments varies significantly from river to river [29]. Although the ecological risk indicators we have measured are low, the long-distance transport, biotic and bioaccumulative effects of PCBs, and the challenges of degradation, make the task of prevention and control more dangerous and are worthy of our attention.

## 4. Conclusions

Based on the current circumstances and historical factors of the Beiluo River, the sediment quality benchmark method (SQGs), potential ecological risk index (RI) and toxic equivalents (TEQs) were selected to evaluate and analyse the pollution status and sources of PCBs in sediments.

(1) Overall, 27 congeners of PCBs were detected in the sediments of the Beiluo River basin, with ∑PCBs ranging from 0.12 to 1.25 ng·g^−1^. PCB concentrations were higher in the upper and lower reaches of the Beiluo River, especially at sampling points B5, B8 and B13. PCBs were predominantly found at each sampling point (>5CL), especially 10-PCBs and 6-PCBs.

(2) The results of the ecological risk assessment of PCBs in the Beiluo River with three methods used demonstrated that PCBs in all sampling sites were slightly contaminated and rarely caused negative biological effects, with a low potential risk to the environment.

(3) The main sources of PCBs in the Beiluo River using the PMF model were technical PCB mixtures, coal combustion and industrial production. The percentages were: 44.93%, 32.46% and 22.61%, respectively. These results provide ideas for pollution control and management at the Beiluo River. However, the samples collected in this study were not comprehensive in terms of time and space, neglecting the differences between field experiments and laboratory environments and the study of bioavailability (the ability of chemicals to be absorbed and possible toxicity). Therefore, more samples and the exact local ecological context should be included to enhance the study of the relevant mechanisms as a way to assess ecological risks more effectively and accurately in future studies.

## Figures and Tables

**Table 1:** Comparisons of PCBs (ng·g^−1^·DW) in sediments from different regions.

Location	Sampling Year	PCB Concentrations	References
Sanya River, China	2019	1.75–92.75	[21]
In and outside Rosetta branch estuary, Egypt	2017	0.98–2.03	[22]
River Thames, England	2016	0.12–27.4	[23]
Pearl River Delta, China	2018	16.15–477.85	[24]
Hooghly River Estuary, India	2018	0.28–7.7	[25]
Delaware River Estuary, USA	2017	18.1–136.8	[26]
Pearl River Estuary, China	2017	4.6–187.4	[27]
Turag River, Bangladesh	2019	1.68–10.6	[28]
Beiluo River, China	2021	0.12–1.25	This study

**Table 2:** Results of pollution assessment for PCBs in the sediments of the Beiluo River using the potential ecological risk index.

Potential risk parameter	E^i^_r_ < 40	40 ≤ E^i^_r_ < 80		80 ≤ E^i^_r_ < 160		160 ≤ E^i^_r_ < 320		E^i^_r_ ≥ 320	
Potential risk level	Slight	Medium		Strong		Very strong		Pole strength	
Sampling points	B1	B2	B3	B4	B5	B6	B7	B8	B9
E_r_	0.57	0.60	0.71	0.80	1.56	0.57	0.52	1.44	0.58
Potential risk level	Slight	Slight	Slight	Slight	Slight	Slight	Slight	Slight	Slight
Sampling points	B10	B11	B12	B13	B14	B15	B16	B17	
E_r_	0.45	0.30	0.48	5.00	1.19	0.49	0.91	0.38	
Potential risk level	Slight	Slight	Slight	Slight	Slight	Slight	Slight	Slight	

**Table 3:** The toxic equivalency factor (TEF) of certain PCBs and their toxicity equivalents (TEQ) (pg·g^−1^).

PCBs	TEF	TEQ	PCBs	TEF	TEQ
PCB-77	0.0001	0.0072	PCB-118	0.00003	0.0016
PCB-81	0.0003	0.0184	PCB-123	0.00003	0.0033
PCB-126	0.1	9.6630	PCB-156	0.00003	0.0020
PCB-169	0.03	1.4784	PCB-157	0.00003	0.0014
PCB-105	0.00003	0.0017	PCB-167	0.00003	0.0008
PCB-114	0.00003	0.0019	PCB-189	0.00003	0.0017

## Data Availability

Not applicable.

## Supplementary Materials

The following supporting information can be downloaded at: https://www.mdpi.com/article/10.3390/toxics11020139/s1, Text 2-2: Sediment handling method; Table S1: Error estimation summary results in PMF model; Table S2: PCB concentration in sediment (dry soil: ng·g^−1^ DW); Table S3: The homolog groups of detected PCBs.
