# Supplementary material for: Distribution, Sources and Risk Assessment of Polychlorinated Biphenyls in Sediments from Beiluo River
Source: Toxics. 2023 Jan 31;11(2):139. doi: 10.3390/toxics11020139 (PMC9958545; doi:10.3390/toxics11020139)
Supplement: Supplementary file 1 [file toxics-11-00139-s001.zip › toxics-2177176-supplementary.pdf]

toxics

Supplementary Materials: Distribution, Sources and Risk Assessment of Polychlorinated Biphenyls in Sediments from Beiluo River

Linrong Han, Chao Chang, Shiwei Yan, Chengkai Qu, Yulu Tian, Jipu Guo, Jiahua Guo

1. Sediment handling method 2-2:

Weighing: Place the sample on an enamelled or stainless steel plate, mix well and remove any foreign matter. Freeze-drying is used. The dried sample needs to be ground and passed through a 150μm (100 mesh) aperture sieve. A 10-20g sample is then weighed for extraction.

Extraction: Accelerated solvent extraction (ASE350) is used.

Concentration: solvent replacement: rotary evaporator, set to a water bath temperature of approximately 40°C and 60 r/min. When the extract in the flask is concentrated to 4 ml, 4 drops of hexane are added. Place the flask in a fume hood with the stopper tightly closed to await chromatography.

Chromatography: Mix dichloromethane and n-hexane in a 2:3 ratio to make an over-column mixture. A suitable amount of skimmed cotton is inserted into the chromatographic column. Fill the column with alumina and silica gel in a 1:2 volume ratio to a total length of approximately 9 cm, with the bottom of the column connected to a chicken heart bottle. Wet and wash the column with about 20 ml of the mixture. When the liquid surface just touches the silica gel surface, pour the liquid collected in the cockle bottle into the waste bottle and quickly connect it back to the bottom of the column, while injecting the concentrated extract from the flask into the chromatography column with a dropper. The flask was washed three times with 3 drops of the mixture at a time and passed through the column in turn. The flask was washed with 25 ml of the mixture.

Re-concentration: The chromatographic solution collected in the cocktail flask is still concentrated to 0.5-1 ml using a rotary evaporator under the same conditions.

Transfer: The liquid in the cocktail flask was transferred to a 2ml cell flask and the flask was washed three times with hexane, the washings were all transferred to the cell flask and the flask was screwed tightly.

Blow nitrogen volume: Place the vial in a nitrogen volume device and blow the solution to 0.2 ml with gentle high purity nitrogen. 5 μl of internal standard (PCNB, PCB141, PCB52) at a concentration of 4 ppm was added for instrumental analysis.

Table S1. Error estimation summary results in PMF model.

Error Estimation				
DISP Diagnostics	Error Code:	0		
	Largest Decrease in Q:	−0.037		
	%dQ:	−0.001643269		
	Swaps by Factor:	0	0	0
BS Mapping	industrial emissions	technical PCBs mixtures	coal and wood combustion	Unmapped
Boot Factor 1	30	0	0	0
Boot Factor 2	0	30	0	0
Boot Factor 3	0	0	29	1

Table S2. PCB concentration in sediment (dry soil: ng·g^{-1}).

**PCBs**	**Mean**	**SD**	**Min**	**Max**	**Det**
PCB-8	0.0045	0.0041	ND	0.0177	31.58%
PCB-18	0.0033	0.0025	ND	0.0090	94.74%
PCB-28	0.0043	0.0049	ND	0.0194	73.68%
PCB-44	0.0032	0.0025	ND	0.0091	89.47%
PCB-66	0.0039	0.0035	ND	0.0139	63.16%
PCB-77	0.0050	0.0063	ND	0.0269	42.11%
PCB-81	0.0036	0.0027	ND	0.0108	94.74%
PCB-101	0.0037	0.0026	ND	0.0107	21.05%
PCB-105	0.0040	0.0045	ND	0.0173	42.11%
PCB-114	0.0048	0.0083	ND	0.0365	36.84%
PCB-118	0.0058	0.0070	ND	0.0298	15.79%
PCB-123	0.0070	0.0094	ND	0.0390	68.42%
PCB-126	0.0070	0.0144	ND	0.0623	31.58%
PCB-128	0.0816	0.1533	0.0007	0.6512	100.00%
PCB-138	0.0071	0.0061	ND	0.0256	94.74%
PCB-153	0.0069	0.0056	0.0017	0.0230	100.00%
PCB-156	0.0039	0.0043	ND	0.0168	94.74%
PCB-157	0.0029	0.0028	ND	0.0107	78.95%
PCB-167	0.0017	0.0017	ND	0.0070	63.16%
PCB-169	0.0030	0.0031	ND	0.0116	73.68%
PCB-170	0.0036	0.0042	ND	0.0141	94.74%
PCB-180	0.0037	0.0072	ND	0.0309	89.47%
PCB-187	0.0055	0.0115	ND	0.0491	78.95%
PCB-189	0.0033	0.0040	ND	0.0155	84.21%
PCB-195	0.0036	0.0042	ND	0.0146	84.21%
PCB-206	0.0088	0.0111	0.0018	0.0458	100.00%
PCB-209	0.0719	0.0314	ND	0.1335	57.89%
PCBs	0.2676	0.2655	0.1236	1.2507	

SD: standard deviation, Det: detection frequencies.

Table.S3. The homolog groups of detected PCBs.

Homolog groups	PCBs	Homolog groups	PCBs	Homolog groups	PCBs
2-PCB	PCB8	5-PCB	PCB101, PCB105, PCB114, PCB118, PCB123, PCB126	8-PCB	PCB195
3-PCB	PCB18, PCB28	6-PCB	PCB128, PCB138, PCB153, PCB156, PCB157, PCB167, PCB169	9-PCB	PCB206
4-PCB	PCB44, PCB52, PCB66, PCB 77, PCB81	7-PCB	PCB170, PCB180, PCB187, PCB189	10-PCB	PCB209
